# Supplementary material for: A method for measuring the distribution of the shortest telomeres in cells and tissues
Source: Nat Commun. 2017 Nov 7;8:1356. doi: 10.1038/s41467-017-01291-z (PMC5676791; doi:10.1038/s41467-017-01291-z)
Supplement: Supplementary file 1 — Supplementary Information [file 41467_2017_1291_MOESM1_ESM.pdf]

a

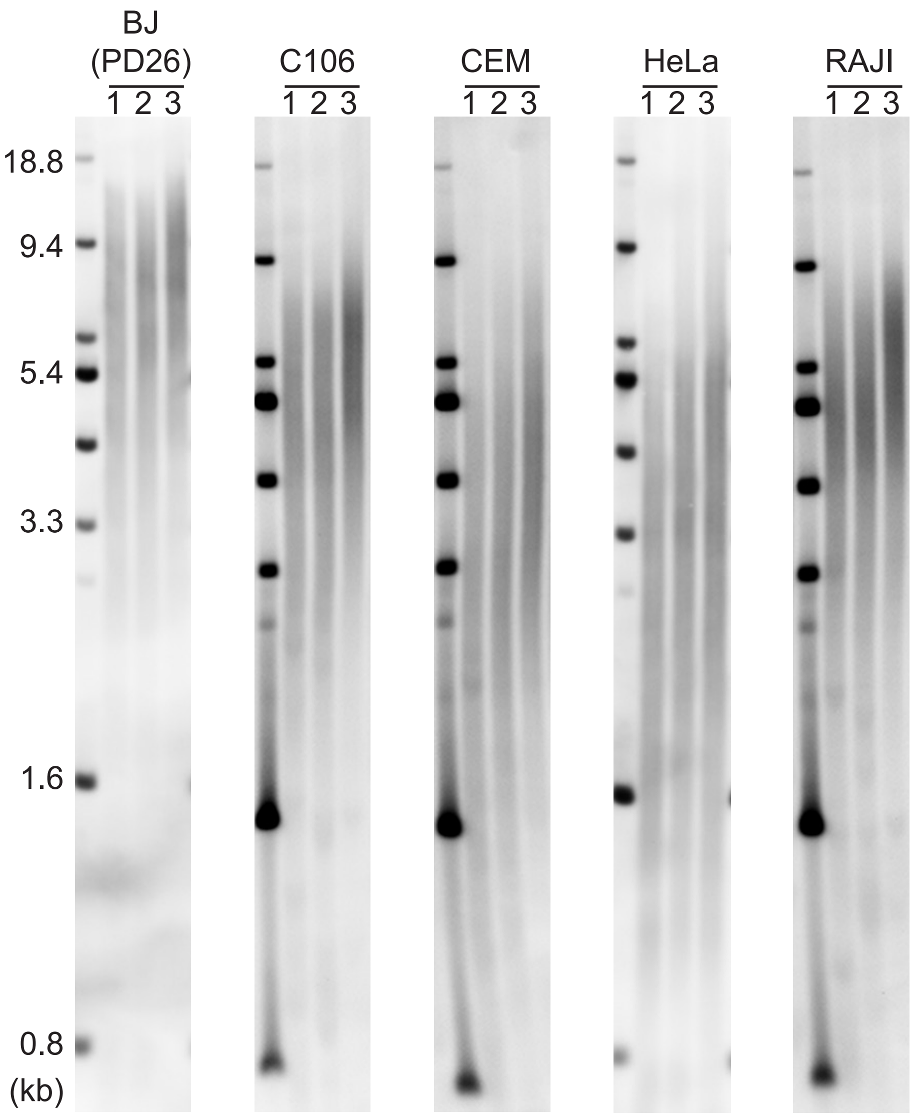
BJ (PD26)
C106
CEM
HeLa
RAJI
1 2 3
18.8
9.4
5.4
3.3
1.6
0.8
(kb)

b

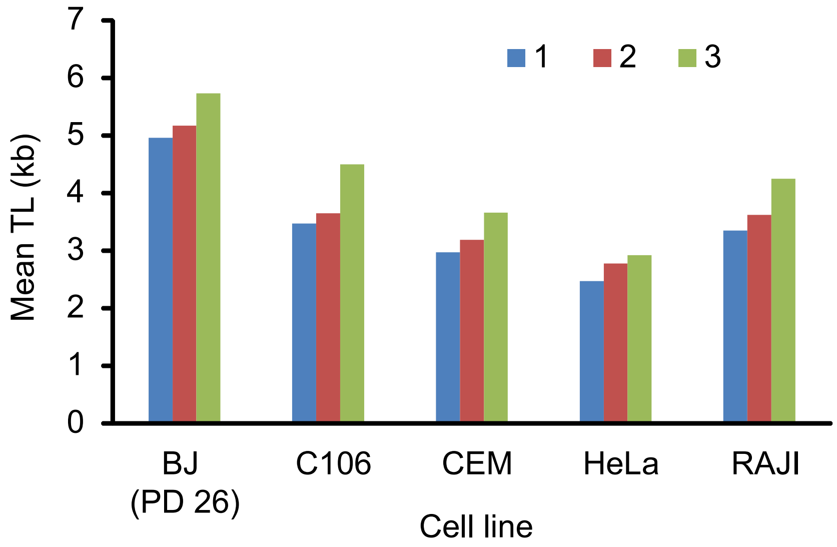
1
2
3
Mean TL (kb)
0
1
2
3
4
5
6
7
BJ (PD 26)
C106
CEM
HeLa
RAJI
Cell line

Supplementary Figure 1. TRF analysis using different combinations of restriction enzymes.

(**a**) DNA of BJ, C106, CEM, HeLA, and RAJI cells were digested with 1, BfaI/CviAII/MseI/NdeI; 2, HphI/MnlI; 3, AluI/HaeIII/HhaI/HinfI/MspI/RsaI. (**b**) Quantification results of each cell line’s TRF analysis in (**a**).

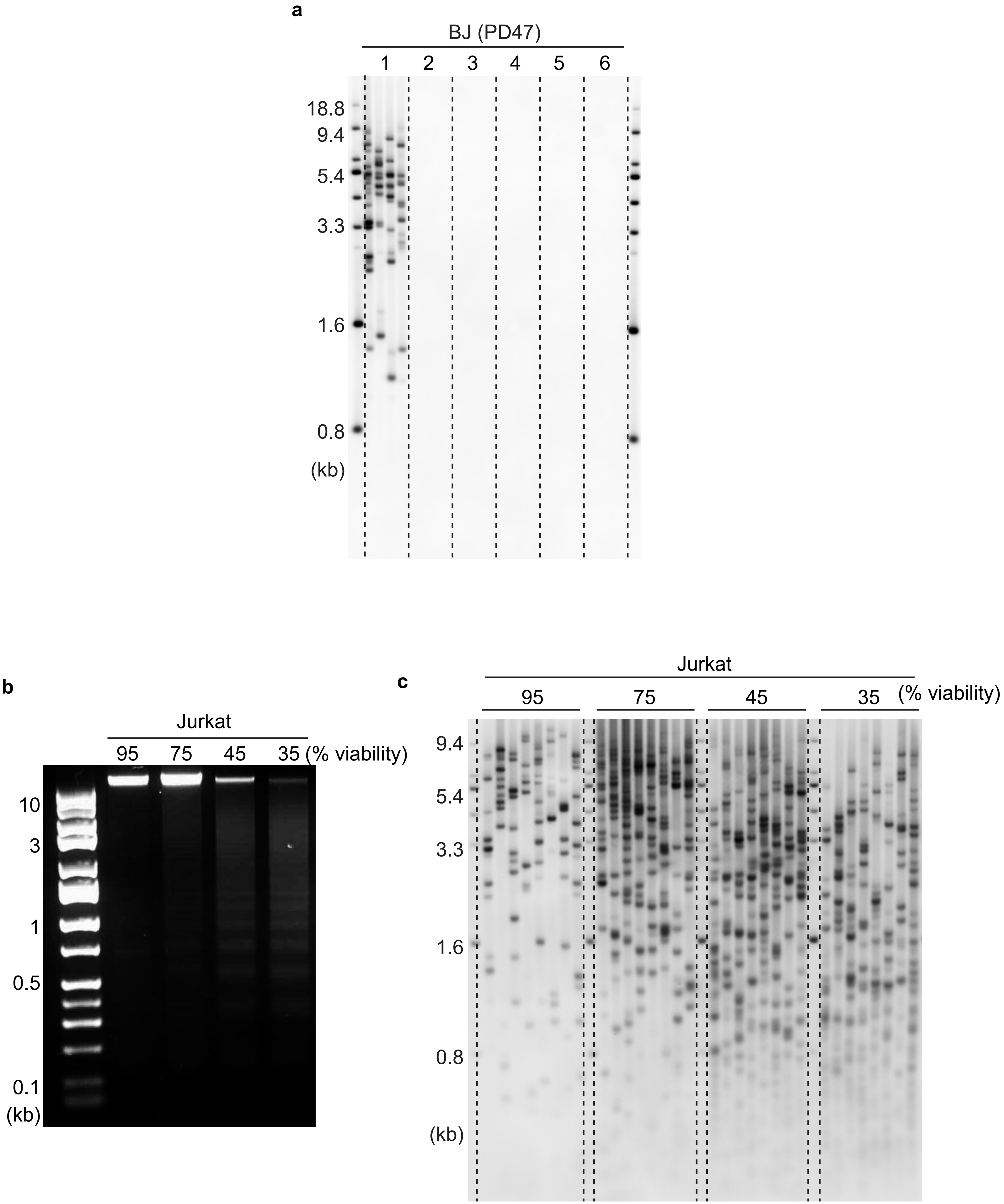
a
BJ (PD47)
1
2
3
4
5
6
18.8
9.4
5.4
3.3
1.6
0.8
(kb)
b
Jurkat
95
75
45
35 (% viability)
10
3
1
0.5
0.1
(kb)
c
Jurkat
95
75
45
35 (% viability)
9.4
5.4
3.3
1.6
0.8
(kb)

Supplementary Figure 2. Validation of TeSLA.

(**a**) DNA from BJ cells was used to perform TeSLA; 1, positive control; 2, negative control without primers for PCR; 3, no TeSLA-Ts for ligations at telomere overhangs; 4, without digestion with REs; 5, no TeSLA adapters for ligations at genomic and subtelomeric DNA; 6, no ligase for any ligation reactions. (**b**) Same amount of genomic DNA (20 ng of each) from Jurkat cells with different viabilities was separated on 1% agarose gel to evaluate DNA integrity. (**c**) TeSLA of Jurkat cells with different percent viable cells.

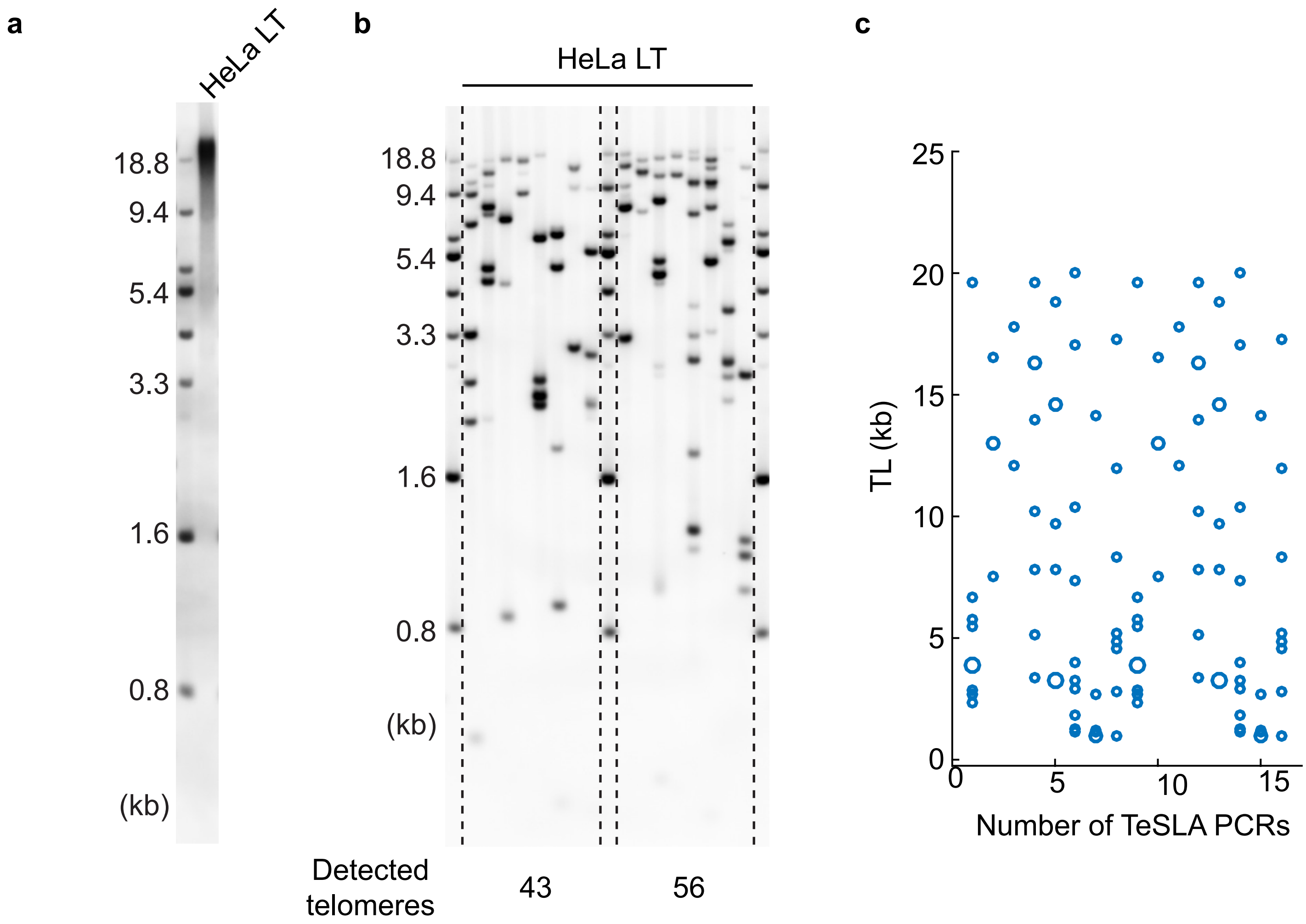

a
HeLa LT
18.8
9.4
5.4
3.3
1.6
0.8
(kb)
b
HeLa LT
18.8
9.4
5.4
3.3
1.6
0.8
(kb)
Detected telomeres
43
56
c
TL (kb)
25
20
15
10
5
0
0
5
10
15
Number of TeSLA PCRs

Supplementary Figure 3. TLs of HeLa LT cells determined by TRF analysis and TeSLA.

(**a**) TLs of HeLa LT cells determined by TRF analysis using the same restriction enzyme combination as TeSLA. (**b**) TeSLA results of HeLa LT (30 pg for each TeSLA PCR reaction, 16 reactions) demonstrated that the upper size limit of TeSLA for TL detection is around 18 kb. (**c**) The scatter plot represents distributions of TLs from TeSLA results (16 reactions) of HeLa LT cells. Each circle represents a particular TL that was detected by TeSLA. The circle size indicates single (small circle) or multiple (large circle) counts for a particular TL.

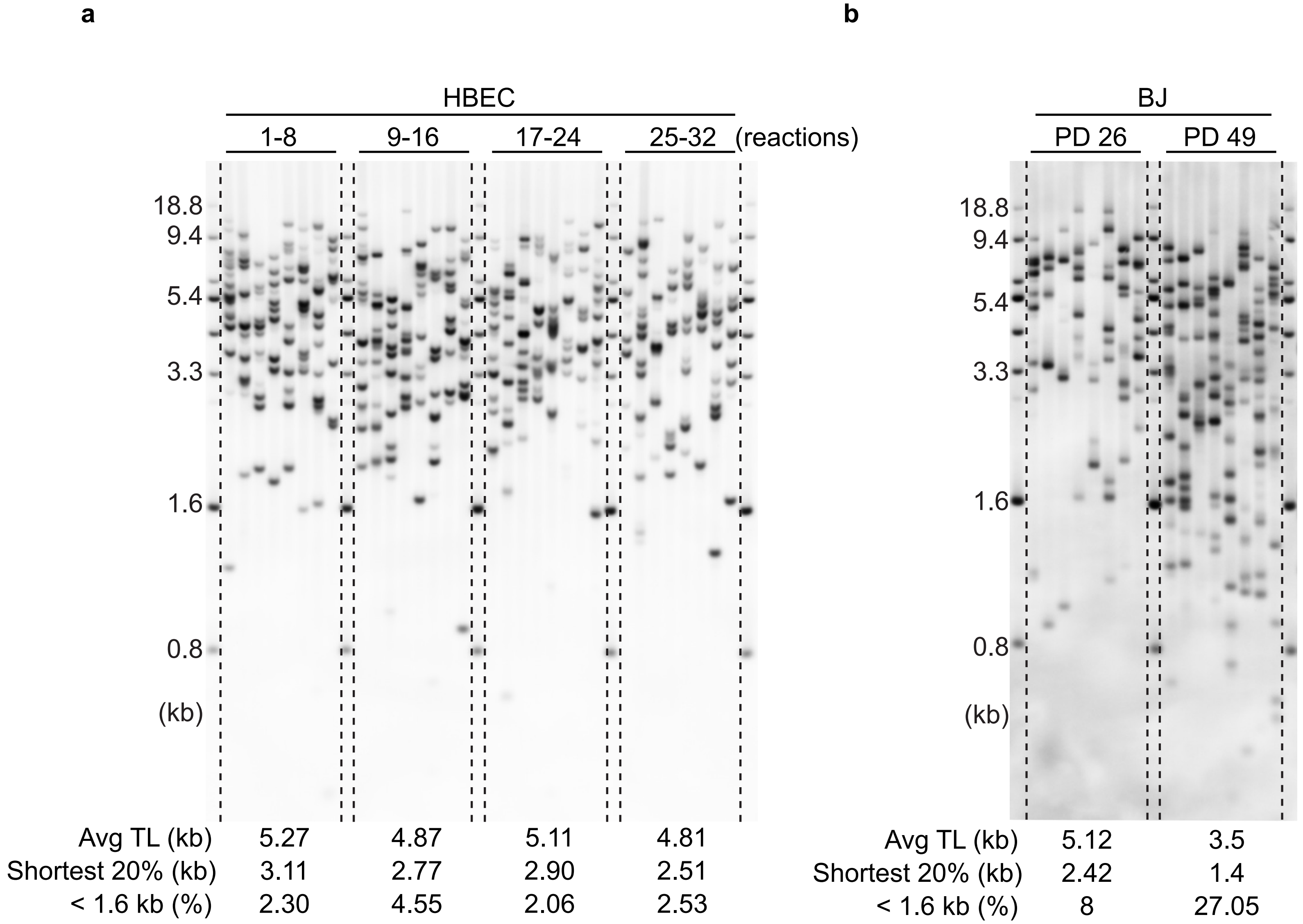
a
HBEC
1-8
9-16
17-24
25-32
(reactions)
18.8
9.4
5.4
3.3
1.6
0.8
(kb)
Avg TL (kb) 5.27 4.87 5.11 4.81
Shortest 20% (kb) 3.11 2.77 2.90 2.51
< 1.6 kb (%) 2.30 4.55 2.06 2.53
b
BJ
PD 26
PD 49
18.8
9.4
5.4
3.3
1.6
0.8
(kb)
Avg TL (kb) 5.12 3.5
Shortest 20% (kb) 2.42 1.4
< 1.6 kb (%) 8 27.05

Supplementary Figure 4. Intra-variation of TeSLA and TLs determined by TeSLA of BJ cells with different PDs.

(**a**) TeSLA results of HBEC (30 pg for each TeSLA reaction, 32 reactions) to determine the intra-variation of TeSLA. (**b**) DNA extracted from different PDs (PD 26 and PD 49) of BJ cells were used to determine TL by TeSLA.

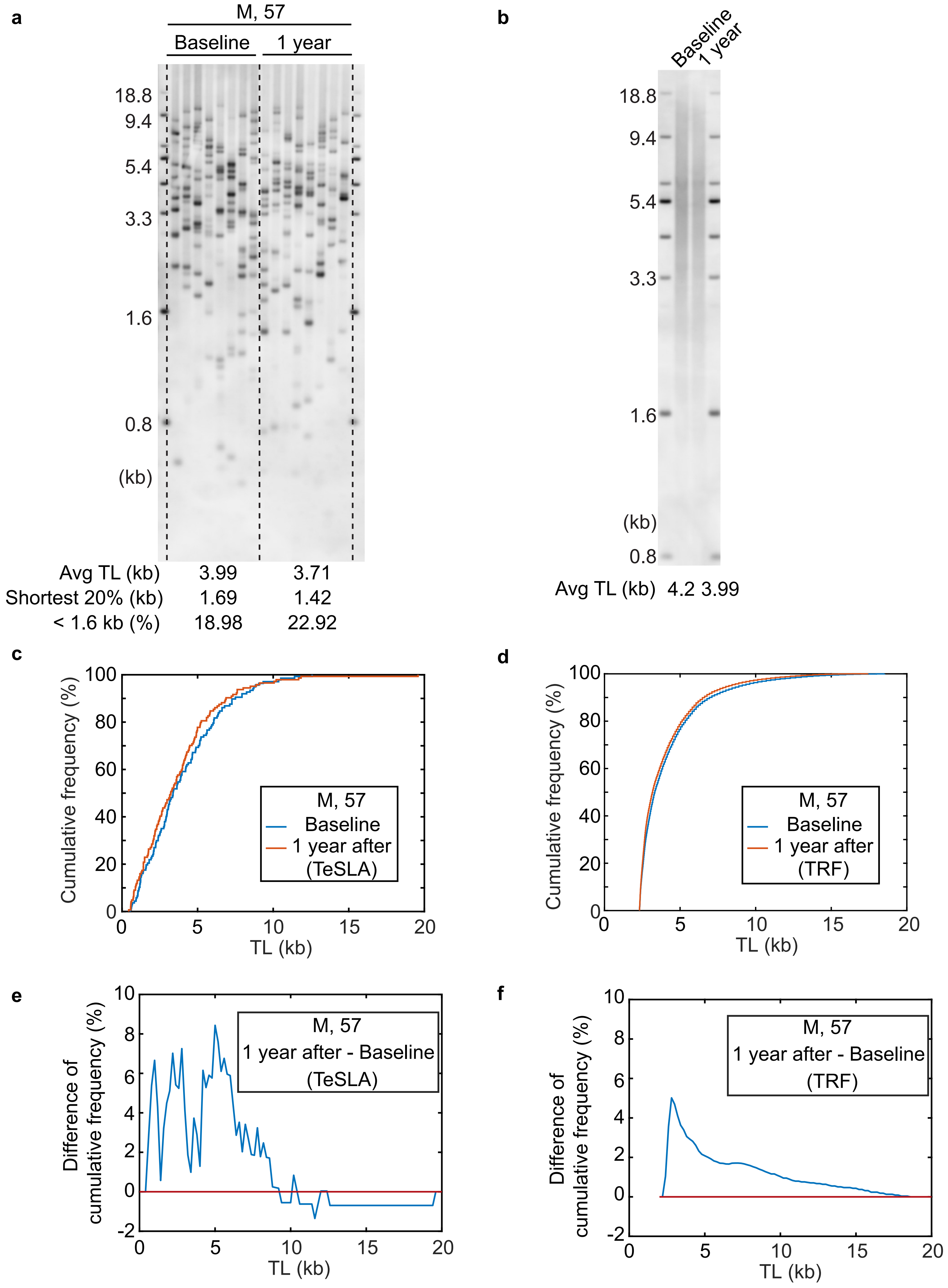

a
M, 57
Baseline
1 year
18.8
9.4
5.4
3.3
1.6
0.8
(kb)
Avg TL (kb) 3.99 3.71
Shortest 20% (kb) 1.69 1.42
< 1.6 kb (%) 18.98 22.92
b
Baseline
1 year
18.8
9.4
5.4
3.3
1.6
(kb)
0.8
Avg TL (kb) 4.2 3.99
c
Cumulative frequency (%)
TL (kb)
M, 57
Baseline
1 year after
(TeSLA)
d
Cumulative frequency (%)
TL (kb)
M, 57
Baseline
1 year after
(TRF)
e
Difference of cumulative frequency (%)
TL (kb)
M, 57
1 year after - Baseline
(TeSLA)
f
Difference of cumulative frequency (%)
TL (kb)
M, 57
1 year after - Baseline
(TRF)

Supplementary Figure 5. Using TeSLA and TRF analysis to determine changes of TLs for human longitudinally over a one year period.

(**a**, **b**) TLs of DNA isolated from PBMCs from a healthy male at age 57 (baseline) and 1 year later were measured by TeSLA (**a**) and TRF analysis (**b**). (**c**, **d**) Empirical distribution curves based on TeSLA (**c**) and TRF analysis (**d**) results to represent TL distributions. The blue (red) lines are TL distributions at baseline (in 1 year after). The increase of cumulative frequency at a TL indicates the effect of TL shortening at the TL. (**e**, **f**) One-year differences in cumulative frequencies as a function of TLs describe one-year change in TL distribution by TeSLA (**e**) and TRF analysis (**f**).

a

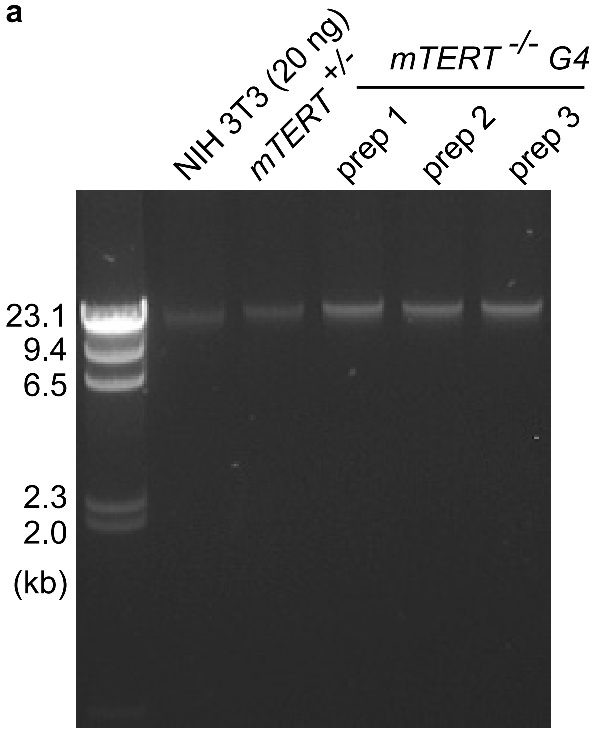

NIH 3T3 (20 ng)
mTERT +/-
mTERT -/- G4
prep 1
prep 2
prep 3
23.1
9.4
6.5
2.3
2.0
(kb)

b

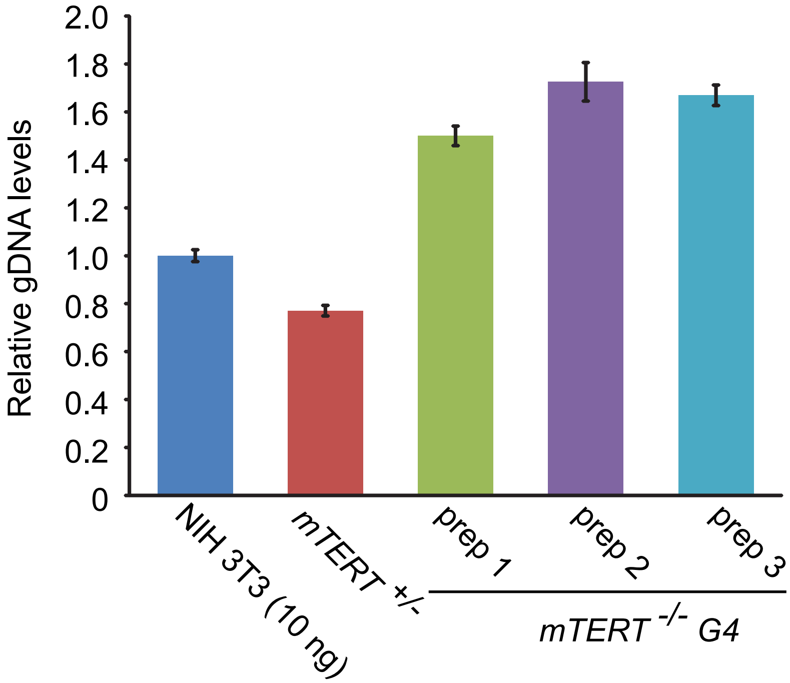

Relative gDNA levels
2.0
1.8
1.6
1.4
1.2
1.0
0.8
0.6
0.4
0.2
0
NIH 3T3 (10 ng)
mTERT +/-
prep 1
prep 2
prep 3
mTERT -/- G4

Supplementary Figure 6. Quality control and q-PCR quantification of extracted DNA from *mTERT*$^{+/-}$ and *mTERT* $^{-/-}$, G4 mice.

(**a**) Extracted genomic DNA from *mTERT* $^{+/-}$ mouse liver tissue, and 3 individual DNA preps from the same *mTERT* $^{-/-}$ mouse liver tissue was separated on 1% agarose gel to evaluate DNA integrity. 20 ng of DNA from NIH 3T3 cells was served as a standard and positive control. (b) Relative DNA levels of *mTERT* $^{+/-}$ and *mTERT* $^{-/-}$ DNA compared with NIH 3T3. Relative DNA levels of each mouse DNA were calculated and normalized to 10 ng of genomic DNA from NIH3T3 cells by q-PCR to amplify mouse B1 repeats. After normalizing DNA concentrations of each sample, the same amount of DNA (50 ng of each) was used to perform TeSLA in **Fig. 7a**. (**b**; mean and s.e.m., n = 3)

Supplementary Table 1.

Oligonucleotides used for TeSLA and mouse genomic DNA quantification.

Oligos for TeSLA	sequence
TeSLA-T1	5’-ACT GGC CAC GTG TTT TGA TCG ACC CTA AC-3’
TeSLA-T2	5’-ACT GGC CAC GTG TTT TGA TCG ATA ACC CT-3’
TeSLA-T3	5’-ACT GGC CAC GTG TTT TGA TCG ACC TAA CC-3’
TeSLA-T4	5’-ACT GGC CAC GTG TTT TGA TCG ACT AAC CC-3’
TeSLA-T5	5’-ACT GGC CAC GTG TTT TGA TCG AAA CCC TA-3’
TeSLA-T6	5’-ACT GGC CAC GTG TTT TGA TCG AAC CCT AA-3’
TeSLA adapter short	5’-GGT TAC TTT GTA AGC CTG TC[SpcC3]-3’
TeSLA adapter TA	5’-[Phos] TAG ACA GGC TTA CAA AGT AAC CAT GGT GGA GAA TTC TGT CGT CTT CAC GCT ACA TT [SpcC3]-3’
TeSLA adapter AT	5’-[Phos] ATG ACA GGC TTA CAA AGT AAC CAT GGT GGA GAA TTC TGT CGT CTT CAC GCT ACA TT [SpcC3]-3’
AP	5’-TGT AGC GTG AAG ACG ACA GAA-3’
TeSLA-TP	5’-TGG CCA CGT GTT TTG ATC GA-3’
**Oligos for mouse genomic DNA**	**sequence**
mB1F	5’-CAG AGG CAG GCG GAT TT-3’
mB1R	5’-GAC AGG GTT TCT CTG TAG CC-3’

[Phos] represents 5’ phosphorylation; [SpcC3] represents C3 spacer
