# Supplementary material for: A method for measuring the distribution of the shortest telomeres in cells and tissues
Source: Nat Commun. 2017 Nov 7;8:1356. doi: 10.1038/s41467-017-01291-z (PMC5676791; doi:10.1038/s41467-017-01291-z)
Supplement: Supplementary file 3 — Description of Additional Supplementary Files [file 41467_2017_1291_MOESM3_ESM.pdf]

Description of Additional Supplementary Files

File Name: Supplementary Software 1
Description: TeSLA-Quant is designed to automatically quantify TeSLA Southern blot images and generate statistical outcomes. It can automatically mark the band positions, detect band intensity, and calculate relevant statistics, such as average size, percentage of short bands, etc. It supports multiple image formats as input. We recommend using tiff as the default input format.
This software gives users the freedom to use different sets of ladders, manually optimize the detection results, and adjust the detection threshold level. It is also able to detect bands on other gel images, such as Western blots and DNA fingerprints.
