# Supplementary material for: A method for measuring the distribution of the shortest telomeres in cells and tissues
Source: Nat Commun. 2017 Nov 7;8:1356. doi: 10.1038/s41467-017-01291-z (PMC5676791; doi:10.1038/s41467-017-01291-z)

TeSLA-Quant User's Manual

August 2017

1. General Introduction

TeSLA-Quant is designed to automatically quantify TeSLA Southern blot images and generate statistical outcomes. It can automatically mark the band positions, detect band intensity, and calculate relevant statistics, such as average size, percentage of short bands, etc. It supports multiple image formats as input. We recommend using tiff as the default input format.

This software gives users the freedom to use different sets of ladders, manually optimize the detection results, and adjust the detection threshold level. It is also able to detect bands on other gel images, such as Western blots and DNA fingerprints.

System Requirements:

TeSLA-Quant is programmed and compiled with Matlab 2016b (Mathworks) and its image processing toolboxes. The required compiler MCR 2016b (9.1) has been incorporated in the installation file 'TeSLAQuant.exe'. Users can simply double click the executable file to install the software in a local computer. The current version is designed for 64-bit Windows 8 or 10 operation system. It may take a few minutes to start the software on computers with low memory or early version systems.

2. Software Input and Output

After the software installation, the user can click ‘Load Image’ to take the input. It requires the 2D single image for each analysis. We provide a sample image for user’s reference. The expectation is to have low background noise, vertically aligned lanes, and one separated lane for ladders.

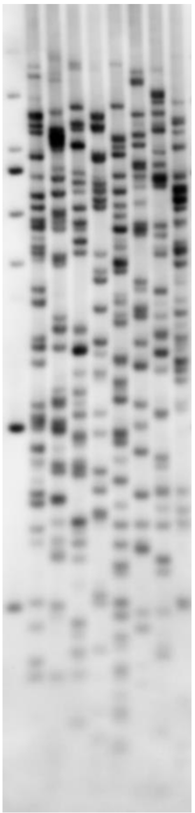

Sample Input

Users can click ‘Save Figure’ after the detection to save the output panel with .jpg format. The software will automatically generate a result folder on the image’s path, which has the same name with the input image, plus a number indicating the analysis time. That folder contains the following output files:

.txt file: Quantification results
.mat file: Matlab variables containing analysis results
.mat file with ‘imageInput’: Input image stored in a 2D matrix
.jpg file: Output software panel
.xlsx file: Excel form containing each band’s intensity and count number

3. Step by Step Guide

1. Load image: Click on the ‘Load Image’ button once the software panel has been launched. The default file format is tiff. The software can remember the user defined path and start and will use the same folder when the next image is to be uploaded.

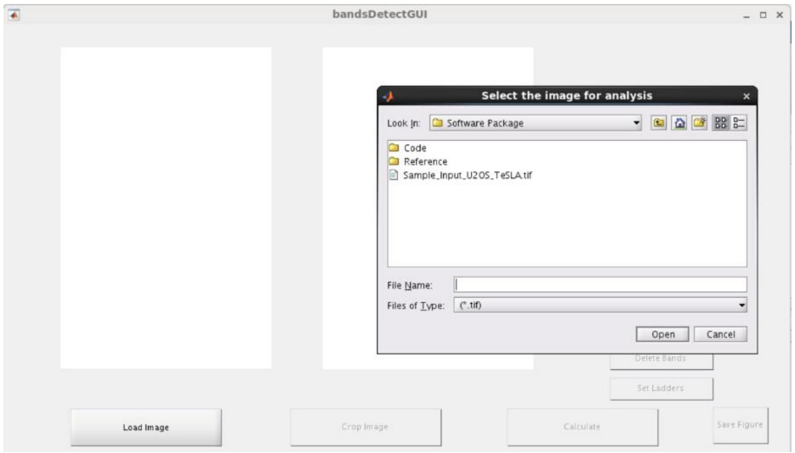

2. Crop image: The user is asked to crop the region of interest. The marker lane needs to be included. User can change the cropped region by dragging the frame and finally confirming the choice by double clicking within the frame.

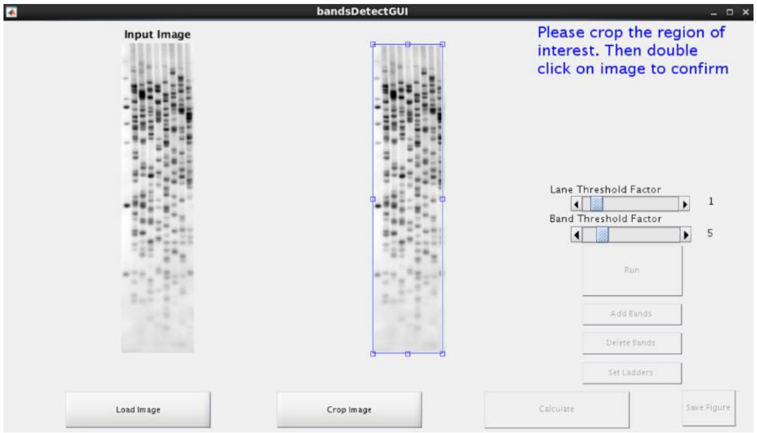

3. Run: The user will get preliminary detection results after clicking the ‘Run’ button. Each red dot marks an individual detected band, including the marker lane. The user will have the freedom to manually adjust band detection results by adding or removing

bands. After clicking on the ‘Add Bands’, the user can click on any suspicious band center to manually add one band. One or multiple bands can also be removed using the ‘Delete Bands’ function. The user needs to click on the image and drag a frame which contains the unwanted bands. Those bands will be removed after user double clicks within the frame. If the image noise level is different from the sample input, the user can adjust the lane/band threshold factor to achieve fine or coarse detection results. The higher number indicates coarser detection power.

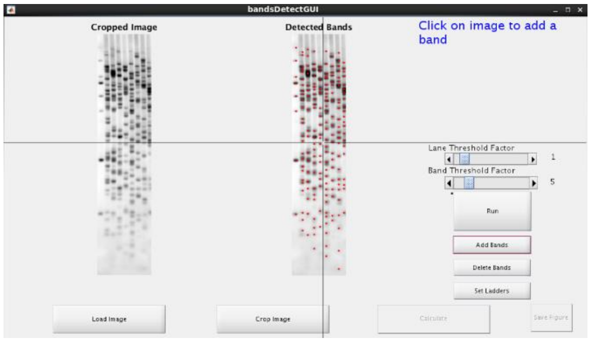

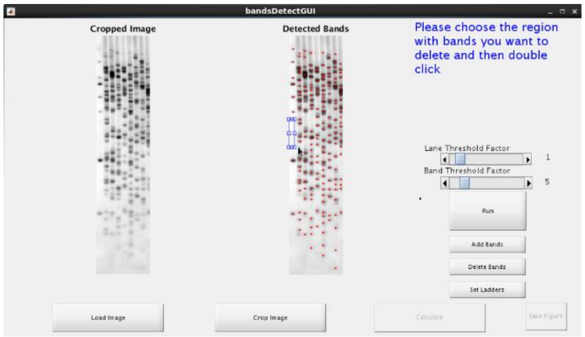

4. Set Ladders: The user needs to click on the image to define the left and right boundary of the ladder lane. The software will report an error message if the user clicks outside the image region. Our default ladder set consists of eight markers from 0.8kb to 18.8 kb. The users are always free to switch to another ladder set as long as they enter ladder number and size accordingly. The software will then fit the ladders with a piecewise log transform regression model and annotate band size by comparing the pixel position of detected bands to ladders.

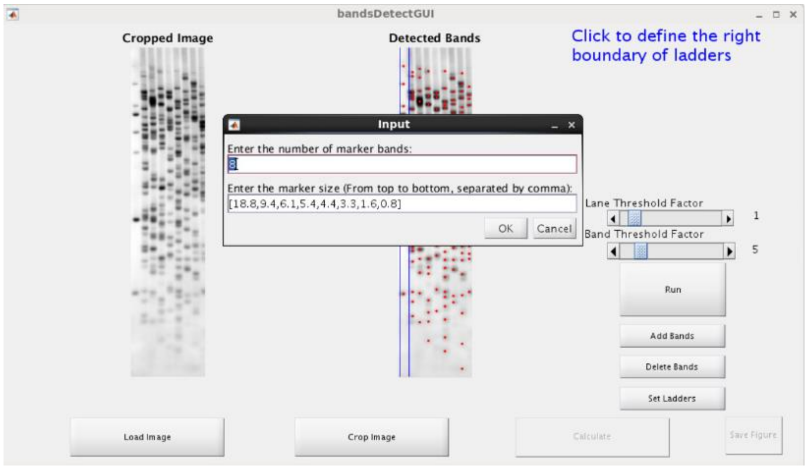

5. Output: The software will give output of average telomere length, ratio of shortest telomeres, and the telomere length of the shortest 20%. Users need to click on the 'Calculate' button and then define the short telomere threshold. The default is 1.6kb. The software will also display the telomere length distribution histogram. To store the final output, a folder with the same name as the input image will be created in the same path. The individual band information will be saved in a mat file. The statistics (e.g. mean, ratio) will be saved in a txt file. The final output interface will be saved in a jpg file if the user clicks 'Save Figure' button. The preprocessed input image will be saved in a mat file as well.

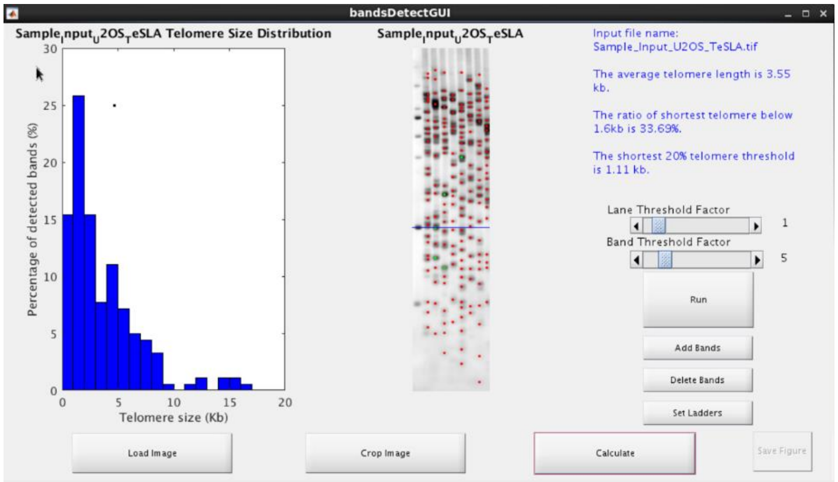

Supplement: Supplementary file 4 — Supplementary Software 1 [file 41467_2017_1291_MOESM4_ESM.zip › Software Package/SoftwareManual.pdf]
