# Supplementary figures and images for: A method for measuring the distribution of the shortest telomeres in cells and tissues
Source: Nat Commun. 2017 Nov 7;8:1356. doi: 10.1038/s41467-017-01291-z (PMC5676791; doi:10.1038/s41467-017-01291-z)

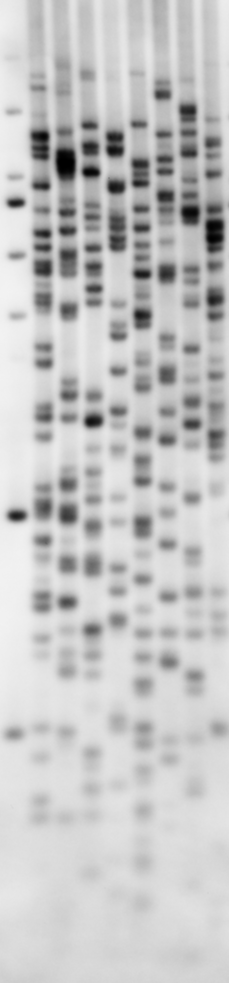

Supplement: Supplementary file 4 — Supplementary Software 1 [file 41467_2017_1291_MOESM4_ESM.zip › Software Package/Sample.tif]
